# From Insecurity to Health Service Delivery: Pathways and System Response Strategies in the Eastern Democratic Republic of the Congo

**DOI:** 10.9745/GHSP-D-21-00107

**Published:** 2021-12-31

**Authors:** Chiara Altare, Vito Castelgrande, Maphie Tosha, Espoir Bwenge Malembaka, Paul Spiegel

**Affiliations:** a Center for Humanitarian Health, Department of International Health, Johns Hopkins Bloomberg School of Public Health, Baltimore, MD, USA.; b Foundation RamaLevina, Bukavu, Democratic Republic of the Congo.; c Center for Tropical Diseases and Global Health, Université Catholique de Bukavu, Bukavu, Democratic Republic of the Congo.; d Department of Epidemiology, Johns Hopkins Bloomberg School of Public Health, Baltimore, MD, USA.

## Abstract

The provinces of North and South Kivu in eastern Democratic Republic of the Congo (DRC) have experienced insecurity since the 1990s. Without any solution to the conflict in sight, health actors have adapted their interventions to maintain some level of health service provision. We reflect on the health system resilience in the Kivu provinces in response to chronic levels of insecurity. Using qualitative interviews of health care providers from local government, United Nations agencies, and international nongovernmental organizations, we identify the mediating factors through which insecurity affects both service quality and delivery and investigate the strategies adopted to sustain service provision.

Three main drivers linking insecurity and health service quality and delivery emerged: via violence, mobility restrictions, and resources availability. The effect of these drivers is mediated by several system or individual-level factors. Two factors were reported in each pathway: health care workforce availability and drug/equipment accessibility. Human resources were affected differently by each driver: in terms of willingness to be stationed in a certain area (violence), capacity to access the health facility (mobility), and sustainability and motivation of conducting duties (resources). Similarly, the presence of drugs/equipment varied in case of looting or damages (violence), delays in delivery (mobility), or delays in procurement (resources). While these mediators are not surprising, their identification allows the design of appropriate response strategies. The majority of the reported solutions attempt to address the lack of human resources and reflect absorptive capacity. Adaptive capacity characterizes the attempts to address lack of access (contingency plan, mobile clinics, maternity waiting homes, and security drugs). Finally, interventions to address insecurity can be classified as transformative. Health actors in eastern DRC have shown some capacity to adapt, adjust, and transform due to insecurity. Further research is needed to measure the effectiveness of such strategies to provide guidance to increasingly vulnerable health systems.

## Abstract (precis)

We identify the mediating factors through which insecurity affects both health service quality and delivery and investigate the strategies adopted to sustain service provision in the provinces of North and South Kivu, Democratic Republic of the Congo.

## Teaser

**Key Findings:**

- Insecurity affects health service delivery and quality in eastern Democratic Republic of the Congo through 3 main pathways: violence, mobility restrictions, and resources availability.
- Of several mediating factors that play a role in service delivery and quality, the 2 most important are health care workforce availability and drug/equipment accessibility.
- Health care providers are implementing a variety of strategies to address such barriers, showing some capacity to adapt, adjust, and transform due to insecurity. Most of the response strategies reflect the absorptive capacity and address the lack of human resources. Adaptive solutions tried to address the lack of access, and transformative interventions address the challenges posed by insecurity.

**Key Implications:**

- Understanding the mechanisms through which insecurity affects health service provision and quality is central to designing appropriate response strategies.
- The effectiveness and cost-effectiveness of response strategies to chronic insecurity should be assessed to provide guidance to increasingly fragile health systems.

Résumé en français à la fin de l'article.

## INTRODUCTION

The provinces of North and South Kivu in eastern Democratic Republic of the Congo (DRC) have experienced insecurity since the early 1990s. Despite a peace agreement in 2002 and elections in 2006, unrest continued with more than 140 armed groups in the region fighting for the control of natural resources and land in 2018.^1^ Communities are highly affected, with almost a quarter of the population classified as in need of humanitarian assistance in 2017 and 2018 (in North Kivu, 2.2 million people and 1.6 million, respectively; in South Kivu, 1.4 and 1.7 million people, respectively).^2^^,^^3^ Hundreds of thousands of people are displaced; in 2017, there were an estimated 1.2 million and 650,000 internally displaced people in North and South Kivu, respectively.^4^

The health situation in North and South Kivu remains dire. Mortality has decreased from the levels characterizing the Congo wars in the 1990s, but both maternal and child mortality remain high (473/100,000 live births in 2017^5^ and 85/1,000 live births in 2019,^6^ respectively). The country is extremely vulnerable to infectious diseases, with multiple concomitant outbreaks occurring in 2020 (Ebola virus disease, measles, cholera, plague, monkeypox, coronavirus disease [COVID-19], and vaccine-derived poliovirus)^7^ as well as a high malaria prevalence. Infectious diseases (such as malaria and TB) remain the main causes of death among both children and adults, exacerbated by a high prevalence of child undernutrition (49.6% and 48% of the children aged under 5 years are stunted in North and South Kivu, respectively).^8^

Health services for women and children are provided by a variety of public, private, national, and international actors attempting to meet population needs.^9^ Coverage of health services varies importantly by service (particularly between basic vs. emergency services)^10^ and between urban and rural areas leading to a varying degree of unmet health needs. Over the years and without any solution to the conflict in sight, health actors working in North and South Kivu have been obliged to learn how to adapt their interventions to the context to maintain an acceptable level of preventive schedulable service coverage.^10^ This seems to be more challenging for emergency services, for which there is limited access by the Kivu population.

Over the years, health actors working in North and South Kivu have had to learn how to adapt their interventions to the context to maintain an acceptable level of preventive schedulable service coverage.

The capacity of a health system to respond adaptively to external shocks that challenge its functioning is a key attribute of a resilient health system and is a common feature across varying definitions of resilience.^11^^–^^14^ A resilient health system aims to minimize service disruptions despite shocks such as conflict, natural disasters, or outbreaks, making it a prerequisite for achieving and sustaining universal health care.^15^ In a context like eastern DRC where the health system faces both acute and chronic challenges, the concept of everyday resilience as an emergent feature of complex adaptive systems^16^ can help frame the adaptative and transformative approaches adopted by the system in response to a changing environment.

As resilience is a dynamic and context-specific process, documenting experiences from different settings contributes to global learning and sharing of experience. In this article, we reflect on the resilience of the health system in North and South Kivu in response to chronic levels of insecurity. We identify the mediating factors through which insecurity affects both service quality and delivery and investigate the strategies that health care providers from local government, United Nations (UN) agencies, and international nongovernmental organizations (NGOs) adopt to sustain service provision. Building upon Blanchet's resilience framework^12^ and looking at the North and South Kivu health system as a complex adaptive system,^16^ we aim to enhance understanding of health system resilience strategies to inform health care providers at national and international levels designing response plans in similar conflict-affected settings.

## METHODS

### Study Design

This analysis is embedded within a broader research project conducted by the BRANCH (Bridging Research and Action in Conflict settings for the Health of women and children) consortium in 10 conflict-affected countries to investigate factors that shape decision making and maternal and child health service delivery.^17^ In DRC, we conducted a mixed-methods case study, using both secondary quantitative and primary qualitative data. More details are provided in the article presenting the results of the case study.^10^ In this substudy, we use qualitative data to identify operational challenges and investigate strategies to maintain service delivery and quality. While service delivery is also influenced by decision making and policy making, we limited the scope of this article to operational aspects to allow for more detailed discussion.

### Study Setting

The case study was conducted in the North and South Kivu provinces in DRC in 2018. North Kivu has experienced higher intensity violence than South Kivu, both in terms of casualties and events. Few fatalities have occurred in South Kivu since 2012, despite numerous violent events throughout the years. Violence against civilians (33.2%) and battles with no change of territory (31.2%) were the most frequent forms of violent episodes in both provinces.^18^

Two health zones in each province were selected due to their history of conflict and insecurity (in terms of active armed clashes, population displacement, and accessibility) during the previous 5 years. The decision was taken in consultation between the research team and representatives of the provincial health offices. In North Kivu, the health zones of Mweso and Ruanguba were visited; in South Kivu, those of Minova and Walungu. Mweso experienced extensive violence, population displacement, and attacks on health facilities; Ruanguba was the center of the March 23 Movement (M23) offensive in 2012–2013; Minova and Walungu have experienced extensive conflict over land issues and customary power.^19^

We selected 2 health zones in each province due to their history of conflict and insecurity during the previous 5 years.

### Data

Qualitative data were collected through individual or group interviews with representatives of private and public health care providers currently working in North and/or South Kivu. These included staff of the Ministry of Health, UN agencies, NGOs, faith-based organizations as well as health care workers (chief midwives, chief nurses, and community health workers). We visited 1 hospital, 1 health center, and 1 health post in each health zone to ensure health facilities of different sizes, services, and resources were included in the study. Two referral hospitals (1 per province) were visited as well.

We conducted 51 in-depth interviews (IDI) and 4 focus group discussions (FGDs), with a total of 84 respondents (Table 1). Participation was voluntary. Oral informed consent was obtained from all participants, who needed to be aged 18 years or older and working in the position for more than 30 days.

**TABLE 1.** Participants in In-Depth Interviews and Focus Group Discussions in Analysis of Insecurity and Health Service Provision and Quality in North and South Kivu Provinces, Democratic Republic of the Congo

In-Depth Interview Participants by Affiliation	North Kivu	South Kivu
Ministry of Health (DPS/MCZ)	4	5
Nongovernmental organization	4	6
United Nations agency	2	2
Health care providers	12	12
Total	22	25
**Focus Group Discussion Participants**		
Community health workers	17 (40% Male; 60% Female)	20 (75% Male; 25% Female)

Abbreviations: DPS, Division Provinciale de Santé (Provincial Health Division); MCZ, Médicin Chef de Zone (Chief Medical Officer of the health zone).

We developed and piloted an interview guide for each respondent group to reflect their role and the mandate of their organizations. Questions aimed to inquire about availability and quality of provided maternal and child health services; factors affecting decision making and program implementation (including human resources, funding, information management, infrastructure, and coordination); challenges and opportunities; adaptations to programs to respond to population displacement and insecurity; and level and type of insecurity in the communities. All interview guides were developed in French; the interview guides for FGDs and facility health care workers were translated into Swahili by the research team members (native speakers). Translation into Swahili was tested during the pilot of the guides and fine-tuned until an agreed-upon formulation was found. A 4-day training of the interviewers was conducted in Bukavu to familiarize them with the project objectives and the data collection tools. The case study coordinator (CA) led the training together with the field research coordinator (MT). Interview guides were piloted in a health facility in Bukavu, which was not included in the sample. Data collection took place between August and September 2018.

### Data Management and Analysis

Interviews took place in French or Swahili according to the preference of the respondent. Recordings were transcribed in French. Data management and coding were done in NVivo.^20^ The codebook included both predefined codes addressing issues the study aimed to explore and additional ones that arose from the interviews. Two team members coded the transcripts after having tested and compared coding approaches to ensure harmonization. Thematic analysis methods were used, whereby data were compiled, disassembled, and then reassembled.^21^ Framework analysis was used to explore data. A matrix output (with cases as row and codes as column) was developed to systematically summarize data and facilitate constant comparison within and across cases and topics.^22^

### Ethics Approval and Consent to Participate

The Johns Hopkins Bloomberg School of Public Health determined that this study was not human subjects research and therefore did not require institutional review board oversight (IRB 8652). In DRC, the study protocol was reviewed and approved by the Université Catholique de Bukavu's Institution Review Board (UCB/CIES/NC/02/2018). Oral consent was obtained from all study participants before initiating data collection.

## FINDINGS

Three main drivers affecting health service delivery and quality were identified from the interviews (Figure): violence, reduced mobility, and resource availability.

**FIGURE:**
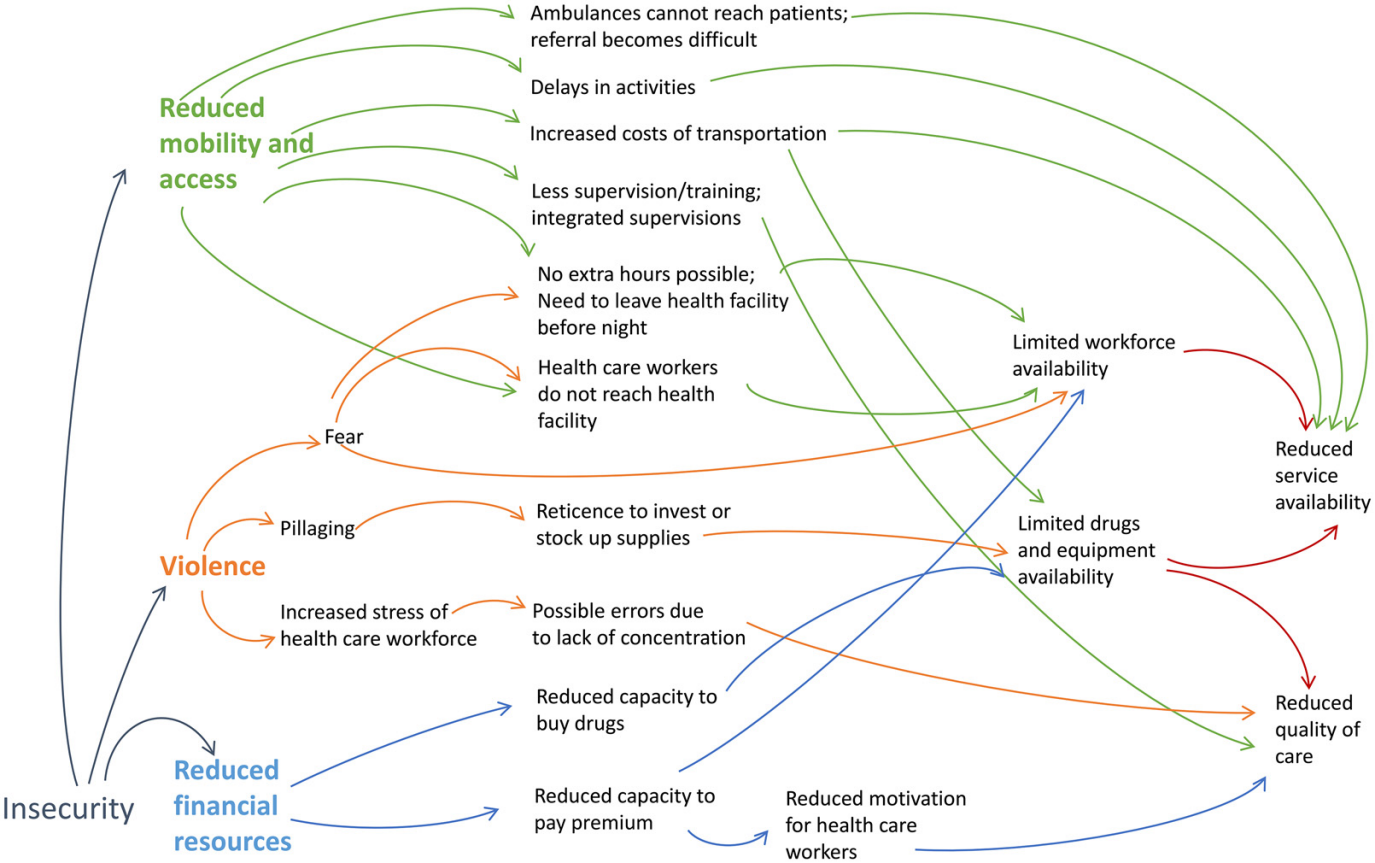
Pathways Through Which Insecurity Affects Health Service Delivery and Quality in North and South Kivu Provinces, Democratic Republic of the Congo

### Driver 1: Overall Insecurity and Attacks on Health Care Workers and Facilities

The first way through which insecurity affects service delivery is through direct or feared violence. This has multiple mediators.

#### Availability of the Health Care Workforce

The occurrence of attacks on health facilities or health care workers often forces health staff to leave the affected area. Partners may be forced to withdraw their presence from the affected area and interrupt activities until security is reestablished. The same is true when the health care workers live with the constant fear that they or their health facilities could be attacked.

*Insecurity affects the availability and quality [of services]; with insecurity qualified health staff flee, we can no longer supply or provide quality care*. —NGO worker

#### Increased Stress

When health care workers decide to stay and work in the insecure area, they report that they often work under stressful conditions comprising fear, restlessness, and instability. These weigh heavily on the psychological health of the staff who reported struggling to concentrate and focus:

*We are not psychologically stable when we are scared; and if we are scared, this can disrupt the care. —Hospital health care provider*

When health care workers decide to stay and work in the insecure area, they report that they often work under stressful conditions comprising fear, restlessness, and instability.

This can increase the risk of committing mistakes or having other lapses in judgment.

*I do not know if there is anybody who has heard a firearm shot and who is in good health, I do not know if such a person exists. If there is insecurity, you will understand, this affects our mentality, it affects our behavior; when the behavior is concerned, are we going to provide quality care? The answer is no.* —Health facility health care provider

*When you know that there is no peace, you can be struck any time, you cannot care for someone, and even if you treat someone, it hits you somewhere … and it limits our actions, our normal reaction to save … you work and you say, “Oh my God, they are going to find me here…” you are somehow traumatized.* —Health facility health care provider

#### Availability of Drugs and Equipment

Participants reported that attacks on health facilities had both direct and indirect consequences. An immediate consequence of the destruction and plundering of health facilities is the reduced availability of functioning equipment and medications.

*… A health center has recently been burgled in [health zone xx] … all material has been plundered; with the plundering of all medicaments if a severe case arrives, there will be no medicament available to treat them, and they can die. This is an effect of insecurity I think.* —FGD participant

In situations of chronic crises like the one in eastern DRC, respondents also reported a sort of “investment reticence,” as the willingness to invest in equipment or supply decreases as the next plundering is around the corner.

*Better not to bring [inputs] as the beneficiaries will not benefit from it; … [the armed groups] will steal and sell it, and when the donor will see the inputs on the market, it will be the partner who will be incriminated*. —NGO worker

*It is not easy to go and look for medicines and (at the same time), we are worried about how to keep the drugs. We cannot buy in great quantity, because you are scared that you will buy for [the armed groups]. I buy in small amounts like a woman going to the market. If I buy in great quantity, they might come and loot.* —Health facility health care provider

### Driver 2: Reduced Mobility and Access of Health Care Workers and Patients

The second challenge identified by the respondents relates to mobility and access constraints that insecurity causes. This works through various pathways.

#### Availability of Health Care Workforce

Health care workers are hindered in reaching the health facilities due to ongoing insecurity either near the facility or along their access route to the facility.

*If the security is threatened, we do not work during the night, we stay at home, and if someone calls you that there is a patient who is turning worse at the hospital, you have to understand that nobody will be available to treat her/him, nobody will put himself in the insecurity to treat a patient.* —Health facility health care provider

If the facility is staffed by visiting clinicians, they may need to abbreviate their service provision hours to travel home before darkness:

*I remember a health facility where they were obliged to close the structure at 3 pm and come back the following day at 8 am, as spending the night there… it meant taking a lot of risks…* —Ministry of Health/Division Provincial de Santé

*…like that Friday, when there were some gunshots in my health area, we started working at noon…*—Health facility health care provider

The same case can be made if there is any form of curfew imposed, either by the government or by the agency providing logistical support.

#### Delays in Reaching Care

Respondents noted that in insecure settings, reaching health care providers can become challenging. Ambulances often struggle to reach patients, and when they do, the route to the health facility may be obstructed. Delays in reaching care also speak to individuals' mobility being limited, either because of the fear of violence or because of limited availability of financial means to pass checkpoints or access ambulance services.

*…Pregnant women delivered under difficult conditions as they didn't have access to the health facilities anymore, thus they preferred deliver at home instead of running the risk of the journey to the health facility.* —NGO worker

*The population takes on a self-management policy with regard to health matters*. —Health facility health care provider

In these scenarios, they tend to resort to home-based solutions such as home deliveries, self-medication, or traditional treatment.

*You will see now, people are resorting to what? You will see people will turn to traditional medicine, they will take herbs, they will take them with the consequence of anemia, and community deaths…* —Health facility health care provider

They will risk the journey to health facilities only in the case of complications or once symptoms of an illness or injury have become too great to bear:

*This does affect us because we have many severe cases, given the security situation, clients arrive late, sometimes they do not arrive at all… this affects us…*—NGO worker

#### Increased Cost of Transportation

When the standard means of accessing health care facilities for both patients and providers are cut off, the use of alternative but higher cost options may be necessary. It was reported that in certain cases, it has been necessary to circumnavigate the insecure areas and travel through Uganda to reach northern parts of North Kivu from Goma. Other times, clinicians needed to access facilities via chartered air services such as helicopters or humanitarian flight services; similarly, patients that require critical care needed to be evacuated by air; and medications, equipment, and material also needed to be transported in the same way. As these methods of transit come at increased costs and require specialized capacity, respondents reported that the number of possible transports is limited, meaning a reduced amount of materials and number of people that can be transported.

*A box of nutrition products which will probably cover [the treatment] of 1 child weighs 15 kg. When you have to pay US$3–4 per kg you see how difficult it is to implement such activity*. —UN agency staff

When the standard means of accessing health care facilities for both patients and providers are cut off, the use of alternative but higher cost options may be necessary.

#### Reduced Training and Supervision

Respondents noted that health staff in conflict-affected areas also may lose access to ongoing training efforts and effective supervision. They highlight the fact that during periods of insecurity, visits from supervisors or training personnel tend to be considered as less essential for the ongoing operations of that health facility. These visits may be interrupted, postponed to more stable times, or conducted remotely. Without being able to directly observe conditions and practice as the health facilities, quality can be compromised:

*It is true, we go there where there is instability, but when there is really insecurity, all what I just told you about our system of regular quarterly visits for the monitoring and monthly for the supervisions, to visit health facilities to ensure service quality, … now, if this is an area where the NGOs [.] can get nowhere, we won't be able to enter the health facility, we cannot really say that we will assure the quality of the provided service* —NGO worker

#### Availability of Drugs and Equipment

In addition to mobility challenges for patients, clinicians, trainers, and supervisors, respondents also noted that access constraints limit their capacity to send necessary supplies and materials in conflict-affected areas. Delays in the delivery lead to increased risk of stock-out of drugs, vaccines, and other medical materials, compromising the capacity to provide treatment and care.

*Yes, this affects the availability of medicines. This means that during insecurity the district office or whoever provides us with medicines cannot pass, how can they reach our position? Thus, there will be stock-outs in the health zone which can easily affect the health center.* —Health facility health care provider

#### Delays in Implementation of Activities

Respondents noted that medical activities may be put on hold during periods of insecurity. Such is the case when mobile clinics are deployed or specialist services are available only at certain scheduled intervals. These activities are often postponed until sufficient levels of security can be assured again, though there is often no set date for the resumption of these services. This has consequences on the overall implementation of work, as well as on the achievement of funding objectives and milestones.

*We need to reassure ourselves of the security conditions, we cannot expose our staff to the risk of death because they want to help. In short, the workplan will not be observed.* —NGO worker

*We have problems with our activity planning. For example, I can plan my visit [to the health facility] taken the inconvenient security context into account. This will delay [everything]… this has the consequence to extend the implementation of activities and even unjustly will influence the results.* —NGO worker

### Driver 3: Resource Availability

The third way in which insecurity affects health service delivery is by the often sudden reduction in patient volume at the health facility level. In a country implementing user fees, such reductions in patients mean that the facility has less revenue available to ensure its normal level of operation:

*Patients are our first chief. With the little amounts they give us, this helps us buying medicaments, paying staff, and having the facility working*. —Health facility health care provider

*Unfortunately, the government's support is close to zero; for this reason, the majority of the staff wishes the number of visits will increase, which is not really the objective of public health (smile).* —Health facility health care provider

In a country implementing user fees, such reductions in patients mean that the facility has less revenue available to ensure its normal level of operation.

The same is true when patients presenting to the health facility are more destitute because of the ongoing conflict and thus have a decreased ability to pay. The facility receives less revenue, but in this case, it also has patients that need care, leading to increasing debts unless specific mechanisms exist that cover costs of vulnerable people (usually provided by external technical and financial partners [TFP]).

More specifically, respondents noted 2 mediators through which reduced patient volume affects service provision and quality.

#### Availability and Motivation of Health Care Workforce

Health facilities use the fees paid by patients to cover the premium for health care workers, which complements the meager salary paid by the government. As these complementary premiums are often what makes staff activity sustainable, payment shortfalls can impact the motivation of the staff to report and provide clinical care, affecting overall availability and quality of care. Retention of health care workers becomes difficult as staff need to look for alternative income sources and tend to look for positions with stable payment (usually those supported by TFP).

*First of all, the system is poorly funded, which causes that if we look at what service providers earn, this does not suffice to cover their survival needs. There, you will find some service providers who are demotivated, and this generates instability of resources. Looking for better (opportunities) elsewhere, health [care] workers run away and desert as well. This means that you can invest in a health provider with training, capacity building, but after a while they will leave and go somewhere else.* —FGD participant

*We can well recruit a skilled health [care] worker, but if we cannot take care of them, at a certain point as we just said that this staff is demotivated, this staff will simply run away. You have to recruit appropriately and ensure an acceptable minimum to stabilize the staff.* —Ministry of Health/Division Provincial de Santé official

Therefore, reduced resources have an immediate impact on service availability and quality but also a broader reduced return of investment of capacity-building activities as turnover risks are high.

Reduced human resources have an immediate impact on service availability and quality but also a broader reduced return of investment of capacity-building activities as turnover risks are high.

#### Availability of Drugs and Equipment

A related consequence of reduced facility revenue is an increased risk of drug stock-outs. Health facilities that are not supported by a TFP use part of the user fees for purchasing their medical supplies and medicines. Reduced revenue leads to less availability both in terms of quantity and variety.

*When utilization decreases, the health facility does not yield, and when it does no longer yield anything, it is difficult to purchase medicines, it is difficult to pay staff, thus the facility has problems in its overall functioning*. —Health facility health care provider

### Strategies to Address the Identified Challenges

Organizations providing services described a variety of strategies they used to overcome the identified challenges. Solutions can be regrouped by addressing 3 areas: lack of human resources, lack of access, and insecurity.

#### Addressing Lack of Human Resources

Respondents reported 4 strategies to compensate for reduced health care workforce.

1. Task shift from higher to lower level or from foreign to national staff. Respondents reported how community health workers are put in charge of needs assessments and management of additional diseases when external teams from the health zone office or the TFP cannot access a given area. Another example of task shifting is substituting a foreign staff person with national staff, who are considered to have easier access in insecure areas.
2. Implement a “self-supervision” model. Registered nurses in charge of a health facility are trained to temporarily take over a supervision role usually attributed to the health zone technical team.

*We trained the chief nurses from each health center on supervision; each one is considered as an internal supervisor and if the Health Zone Central Office team doesn't arrive, the chief nurse acts officially as internal supervisor. Secondly, we trained the supervisors from the central offices of the health zone, so when the team from [NGO] is prevented from being there, the supervisors from the health zone do the supervision. Therefore, we have strengthened the system in such a way that if we cannot be there or are prevented from being there, we know they can still do something to ensure quality services.* —NGO worker

3 Rapidly train agents, or nurses, who have been trained only for a specific task but are then given additional tasks but without having received complete training.
4 Train a pool of staff (instead of only 1 person) to anticipate the high turnover.

#### Addressing Lack of Access

Respondents described 4 strategies used to compensate for the reduced mobility and access.

1. Implement a contingency plan that limits field presence and movements and therefore the exposure of humanitarian staff to insecurity. This was mainly reported by NGOs, rather than government health care workers.
2. Use mobile clinics to bring a set of health services to communities when individuals have difficulties in reaching the health center.
3. Establish maternity waiting homes (known as "Binyolas") to allow close-term pregnant women to stay within the health facility while awaiting the onset of labor. Given the current pattern of armed conflict in the Kivu mostly characterized by one-off attacks or clashes between armed groups rather than prolonged military sieges, during phases/days of relative peace between armed clashes, a woman may decide to go to the nearest health facility with a maternity waiting area until the onset of labor.
4. Distribute preventive drugs both at the facility level and patient level. In certain cases (and specifically for chronic diseases), some organizations give out “security drugs,” (i.e., supply of medications for a longer period so that patients can go to the health facility less frequently).

*For example, in our TB or HIV program, we give out “security medicines”: it is usually necessary for a patient to come every month to get drugs, but we give 3 months because of insecurity. This is specific when it is needed to flee, and you do not have the possibility to go back for medicines.* —NGO worker

#### Addressing Insecurity

Besides operational challenges, organizations also implement activities to address overall security and acceptance at 2 levels. At the community level, organizations aim to strengthen acceptance and facilitate the implementation of activities through dialogues and engagement with different population groups. This entails working with community members to establish an appropriate way to gather, share, and use information about community needs and organization's activities and find an acceptable way to implement programs. Humanitarian organizations have also developed codes of conduct, agreements, and operational standards to ensure they are accountable to the population they serve.

*For security, yes, the overall strategy that we use is to promote acceptance which is our trademark in our communities, and we ensure […] that our programs are [of] high quality; that the promises we make to our beneficiary population are kept; we provide information first to the population and to the partners; for each commitment, we sign a protocol and we make every effort to respect this protocol; […] we have codes of conduct for the staff, for our partners ensuring that we don't do anything foolish in the community, codes of conduct linked to sexual life, to protection, against exploitation and sexual abuse, code of conduct against fraud, fraud sensitivity, and all that, because there are stories like that which once started create problems down the road.* —NGO worker

The second strategy is advocating and increasing awareness at national and international levels by tracking attacks on health care and advocating for safe humanitarian space. A specific human resource is usually appointed as security focal point or security advisor who monitors the situation, approves movements, and advises on risk-management strategies.

## DISCUSSION

Three main pathways linking insecurity and health service delivery and quality in eastern DRC emerged from the study participants' recounts: via violence, mobility restrictions, and resources availability. The effect of these drivers is mediated by several system-level or individual-level factors (Table 2). Two of these factors (health care workforce availability and drug/equipment accessibility) were reported in each of the pathways, highlighting both their centrality and their vulnerability for health service delivery and quality. Human resource availability was affected differently by each driver: in terms of willingness to be stationed in a certain area (violence), in terms of capacity to access the health facility (mobility), and in terms of sustainability and motivation of performing the duties (resources). Similarly, the presence of drugs/equipment in a health facility could vary in case of looting or damages (violence), delays in delivery (access), or delays in procurement due to lack of funding (resources). While these mediators are not surprising and in line with other conflict-affected settings,^13^^,^^23^^,^^24^ their identification can inform response strategies.

**TABLE 2.** Drivers and Mediators of the Links Between Insecurity and Health Service Provision and Quality in North and South Kivu Provinces, Democratic Republic of the Congo

Level	Mediators	Drivers		
		Violence	Mobility/ Access	Resources
Health system	Health care workforce availability	X	X	X
	Drugs and equipment availability	X	X	X
	Delays in reaching care		X	
	Transportation costs		X	
	Training and supervision		X	
	Delays in implementing activities		X	
Individual	Stress	X		
	Reduced motivation			X

Outlining drivers and pathways is conceptually useful as it allows us to untangle the more complex dynamics that shape reality and provides insight into the mechanisms behind outcomes. However, a linear interpretation of such processes is too simplistic as it does not recognize that processes are shaped by the interaction of many system components, which (both the interaction itself and the components) vary according to the context, the severity of the challenges, the actors involved, competing priorities, and other external factors. Looking at the health system as a complex adaptive system allows us to acknowledge the pathways and related solutions while embracing uncertainty, the uniqueness of the context, and emergent characteristics.^25^ Adaptation, learning, and flexibility to emerging issues are even more relevant in humanitarian crises which are precarious by nature.

Outlining drivers and pathways is conceptually useful as it allows us to untangle the more complex dynamics that shape reality and provides insight on the mechanisms behind outcomes.

Resilience has been recognized as an emergent property of complex adaptive systems, arising from the combination of absorptive, adaptive, and transformative strategies.^26^ The majority of the solutions reported by respondents attempt to address the lack of human resources. Applying Blanchet's approach,^12^ these can mainly be classified as absorptive strategies aimed at maintaining service delivery using the same level of resources and capacity. Task shifting, the expansion of job descriptions, and the increased reliance on community health workers has been used in several conflict-affected settings including Syria, Somalia, Uganda, and Sierra Leone^13^^,^^24^^,^^27^ with positive results. However, permanent insecurity often makes accountability, quality assurance, and health system regulation difficult, challenging the capacity to ensure that such adaptations do not turn into lucrative opportunities or lead to lower standards of service provision. Anecdotal examples include registered nurses running health facilities while appointed medical doctors provide more lucrative surgical activities or run parallel private facilities in safer areas or nurses conducting surgeries and cesarean deliveries (despite not being trained for this) as they are the only health professional available in a given area. While this article focuses on insecurity, the lack of financial and human resources and related strategies to overcome it also characterizes broader fragile settings, where the government role may be limited affecting recruitment, retention, and distribution of health care workers.^28^

While adaptive capacity was shown in only 1 human-resource-related solution, it characterizes all attempts to address lack of access. In fact, the activation of a contingency plan, the use of mobile clinics, maternity waiting homes, and the introduction of “security drugs” show the capacity of health care providers to adjust to the new situation and provide alternative ways to maintain service delivery. Mobile clinics are extensively used in humanitarian settings to increase equity in access despite their comparatively high cost, complexity, and inefficiency.^29^ Yet, they remain at times the only feasible way to reach communities. Runaway bags ensure treatment continuity by reducing the risk of interruption due to abrupt service disruption. They can therefore represent a temporary solution in times of insecurity and are best suited for chronic conditions such as HIV^30^ or TB.^31^ Community health workers are playing an increasing role in ensuring continuity of health service provision as well as monitoring and evaluation of health programs (through community-based data collection systems for instance), particularly in areas affected by conflict.^32^

Lastly, the interventions aimed at addressing insecurity can be classified as transformative, as they introduce approaches that would not be necessary without insecurity or are designed to address a specific consequence of insecurity. While community engagement is (or should be) standard practice in the design and implementation of any program to ensure relevance, acceptability, sustainability, and overall success, community dialogues in insecure settings become mechanisms to ensure safety and security of humanitarian workers and communities and require specific negotiation skills that consider conflict, history, and power dynamics. Similarly, the need to establish a mechanism to track attacks on health care illustrates the capacity to integrate a new function in the system (i.e., advocacy for the end of such attacks and of the perpetrators' impunity).

Community dialogues in insecure settings become mechanisms to ensure safety and security of humanitarian workers and communities and require specific negotiation skills that consider conflict, history, and power dynamics.

While lack of resources has been reported as one of the main challenges for health facilities, this tends to apply to health facilities that rely solely on governmental funding. Conflict-affected health zones have received greater amounts of funding and external support in the form of humanitarian aid compared to the least insecure zones.^33^ This led to a 2-tiered system where health facilities supported by TFP have the capacity to provide more and better services, while health facilities relying on out-of-pocket expenses are faced with volatile resources and therefore struggle to ensure the continuity of services.^10^

Insecurity was reported to affect service provision and quality by impacting both the system as a whole and the health care workers as individuals. Stress, fear, and reduced motivation can compromise health service quality even when equipment and medicines are available. Yet, no intervention aimed at providing psychological support to health care workers was mentioned by the respondents. Individual strategies seemed rather to rely on personal religious values or sense of duty and attachment to the community (results not shown). Studies on how health care workers experienced and responded to conflict showed that a resilient health care workforce can contribute to the overall resilience of the system.^24^ However, they also highlighted the possible dissonance between individual and system level-coping strategies (such as dual practice or taking on other jobs outside the health system to complement salary).

### Limitations

This study has several limitations. First, our study reported on challenges in health service delivery as perceived and experienced by service providers only. Although we interviewed actors that have different roles along the “service delivery chain” (i.e., national authorities, donors, and implementers, including both national and international project staff and health care workers) to triangulate findings and obtain a comprehensive understanding of the situation, because we did not include beneficiaries among the respondents, the communities' perspectives on quality of services and actual access could not be presented. Unfortunately, this was beyond the scope of the BRANCH project and the research team could not influence this decision. Our analysis thus provides a partial view of the challenges to increasing health service coverage and quality. Second, the discussed solutions only reflect the experience of the interviewed organizations and are not based on a quantitative assessment of the relative effectiveness or cost-effectiveness of each solution. As evaluating the effectiveness of such efforts was beyond the scope of this study and of the BRANCH overarching project, further research is needed to assess the absolute and relative performance of the proposed solutions. Finally, 1 of the originally targeted health zones in North Kivu (Beni) was not accessible at the time of qualitative data collection due to the Ebola epidemic. Therefore, another health zone was selected among the most affected by violent events.

## CONCLUSION

We used interviews from health care providers to analyze how insecurity affects the delivery and the quality of maternal and child health services. The 3 main drivers are violence, mobility restrictions, and financial resources availability, but numerous mediators have been identified. Understanding the mechanisms behind outcomes allows the design of appropriate response strategies. Yet, no process is linear, rather it is influenced by the uniqueness of the context as well as the uncertainty characterizing humanitarian settings. Health actors in eastern DRC have shown some capacity to adapt, adjust, and transform due to insecurity. However, further research is needed to measure the effectiveness of such strategies to provide guidance for increasingly vulnerable health systems.

## Translation

**En Français**

**De l'insécurité à la prestation de services de santé: voies et stratégies de réponse du système dans l'est de la République démocratique du Congo**

**Résumé**

Les provinces du Nord et du Sud Kivu dans l'est de la République démocratique du Congo (RDC) connaissent l'insécurité depuis les années 1990. Sans aucune solution au conflit en vue, les acteurs de la santé ont adapté leurs interventions pour maintenir un certain niveau de prestation de services de santé. Nous réfléchissons à la résilience du système de santé dans le Kivu face aux niveaux chroniques d'insécurité. À l'aide d'entretiens qualitatifs avec des prestataires de soins de santé du gouvernement local, des agences des Nations Unies et des organisations non gouvernementales internationales, nous identifions les facteurs médiateurs par lesquels l'insécurité affecte à la fois la qualité et la prestation des services et étudions les stratégies adoptées pour soutenir la prestation des services.

Trois principaux facteurs reliant l'insécurité à la qualité et la prestation des services de santé ont émergé: via la violence, les restrictions de mobilité et la disponibilité des ressources. L'effet de ces moteurs est médié par plusieurs facteurs au niveau du système ou au niveau individuel. Deux facteurs ont été signalés dans chaque voie: la disponibilité du personnel de santé et l'accessibilité aux médicaments et à l'équipement. Les ressources humaines ont été affectées différemment par chaque conducteur: en termes de volonté d'être stationné dans une certaine zone (violence), de capacité à accéder à la structure de santé (mobilité), et de durabilité et de motivation des tâches (ressources). De même, la présence de médicaments/équipements variait en cas de pillage ou de dégâts (violence), de retard de livraison (mobilité), ou de retard d'approvisionnement (ressources). Bien que ces médiateurs ne soient pas surprenants, leur identification permet de concevoir des stratégies de réponse appropriées. La majorité des solutions signalées tentent de remédier au manque de ressources humaines et reflètent la capacité d'absorption. La capacité d'adaptation caractérise les tentatives pour remédier au manque d'accès (plan d'urgence, cliniques mobiles, maisons d'attente de maternité, médicaments de sécurité). Enfin, les interventions visant à lutter contre l'insécurité peuvent être qualifiées de transformatrices. Les acteurs de la santé dans l'Est de la RDC ont montré une certaine capacité d'adaptation, d'ajustement et de transformation en raison de l'insécurité. Des recherches supplémentaires sont nécessaires pour mesurer l'efficacité de telles stratégies afin de fournir des orientations aux systèmes de santé de plus en plus vulnérables. La capacité d'adaptation caractérise les tentatives pour remédier au manque d'accès (plan d'urgence, cliniques mobiles, maisons d'attente de maternité, médicaments de sécurité). Enfin, les interventions visant à lutter contre l'insécurité peuvent être qualifiées de transformatrices. Les acteurs de la santé dans l'Est de la RDC ont montré une certaine capacité d'adaptation, d'ajustement et de transformation en raison de l'insécurité. Des recherches supplémentaires sont nécessaires pour mesurer l'efficacité de telles stratégies afin de fournir des orientations aux systèmes de santé de plus en plus vulnérables. La capacité d'adaptation caractérise les tentatives pour remédier au manque d'accès (plan d'urgence, cliniques mobiles, maisons d'attente de maternité, médicaments de sécurité). Enfin, les interventions visant à lutter contre l'insécurité peuvent être qualifiées de transformatrices. Les acteurs de la santé dans l'Est de la RDC ont montré une certaine capacité d'adaptation, d'ajustement et de transformation en raison de l'insécurité. Des recherches supplémentaires sont nécessaires pour mesurer l'efficacité de telles stratégies afin de fournir des orientations aux systèmes de santé de plus en plus vulnérables.

**Principales conclusions**

- L'insécurité affecte la prestation et la qualité des services de santé dans l'est de la RDC à travers 3 voies principales: la violence, les restrictions de mobilité et la disponibilité des ressources.
- Plusieurs facteurs médiateurs jouent un rôle: les 2 plus importants sont la disponibilité du personnel de santé et l'accessibilité aux médicaments/équipements.
- Les prestataires de soins de santé mettent en œuvre diverses stratégies pour surmonter ces obstacles, montrant une certaine capacité à s'adapter, à s'ajuster et à se transformer en raison de l'insécurité. La majorité des stratégies d'intervention reflètent la capacité d'absorption et traitent le manque de ressources humaines; des solutions adaptatives ont tenté de remédier au manque d'accès et des stratégies de transformation aux défis posés par l'insécurité.

**Implications clés**

- Comprendre les mécanismes par lesquels l'insécurité affecte la prestation et la qualité des services de santé est essentiel pour concevoir des stratégies de réponse appropriées.
- L'efficacité et la rentabilité des stratégies de réponse à l'insécurité chronique doivent être évaluées pour fournir des orientations aux systèmes de santé de plus en plus fragiles.

**Peer Reviewed**
